# A ruptured internal carotid artery aneurysm located at the origin of the duplicated middle cerebral artery associated with accessory middle cerebral artery and middle cerebral artery aplasia

**DOI:** 10.4103/2152-7806.69378

**Published:** 2010-09-16

**Authors:** Naoki Otani, Hiroshi Nawashiro, Nobusuke Tsuzuki, Hideo Osada, Takamoto Suzuki, Katsuji Shima, Kanji Nakai

**Affiliations:** Department of Neurosurgery, National Defense Medical College, Saitama, Japan

**Keywords:** Subarachnoid hemorrhage, duplicated middle cerebral artery, accessory middle cerebral artery, cerebral aneurysm

## Abstract

**Background:**

Intracranial vascular anomalies involving the middle cerebral artery (MCA) are relatively rare, as such knowledge will be helpful for planning the optimal surgical procedures.

**Case Description:**

We herein present the first case of a ruptured internal carotid artery aneurysm arising at the origin of the hypoplastic duplicated MCA associated with accessory MCA and main MCA aplasia, which was revealed by angiograms and intraoperative findings.

**Conclusion:**

In practice, this case highlights the urgent need to preoperatively recognize such vascular anomalies as well as understand the collateral blood supply in cerebral ischemia associated with these MCA anomalies.

## INTRODUCTION

Intracranial vascular anomalies involving the middle cerebral artery (MCA) are relatively rare. Teal *et al*.[6] established a distinction between two types of accessory MCA (acc-MCA). The vessel arising from the distal internal carotid artery (ICA) between the anterior choroidal artery and the terminal bifurcation of the ICA, and feeding the vascular territory of the normal MCA, is referred to as a duplicated MCA (dup-MCA). The dup-MCA supplies the cortical region of the temporopolar and the anterior or middle temporal arteries. On the other hand, a vessel that originates between the A1 and proximal A2 segments of the anterior cerebral artery (ACA) reaches the sylvian fissure and feeds the region of the MCA, and is defined as an acc-MCA.[6,9] Although many cases have been reported with one of these MCA anomalies, the coexistence of dup-MCA and ipsilateral acc-MCA with a cerebral aneurysm is extremely rare. In this report, we describe the first case of a ruptured ICA aneurysm arising at the origin of a dup-MCA associated with an acc-MCA and main MCA aplasia.

## CASE REPORT

**Figure 1:**
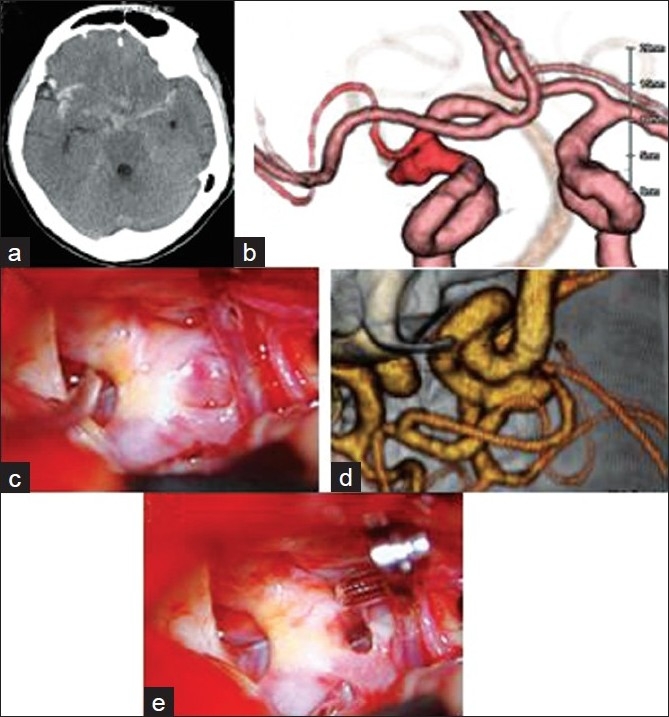
(a) CT scan on admission revealed a diffuse SAH with laterality on the right Sylvian fissure. (b and d) A 3D-CTA showed an ICA aneurysm located at the origin of hypoplastic dup-MCA associated with an acc-MCA arising from the anterior cerebral artery to share in supplying the right MCA territory. (c) The aneurysm measured 6 mm in size and was directed laterally. An intraoperative photograph showing the dup-MCA, the acc-MCA, and the A1 segment of the anterior cerebral artery. After the dissection of the carotid cistern, we traced the right ICA, and observed that the dup-MCA arose from the proximal ICA with an aneurysm arising from the trunk of these vessels. (e) The dup-MCA projected toward the Sylvian fissure. With the further retraction of the frontal lobe, aplasia of the main MCA was confirmed. The neck of the aneurysm was thereafter dissected and clipped successfully

A 66-year-old female suffered a sudden onset of headache and a loss of consciousness. On admission, a computed tomography (CT) scan revealed a diffuse subarachnoid hemorrhage (SAH) with laterality on the right Sylvian fissure [Figure 1a]. A three-dimensional CT angiograph (3D-CTA) showed an ICA aneurysm located at the origin of the hypoplastic duplicated middle cerebral artery (dup-MCA) associated with an accessory middle cerebral artery (acc-MCA) arising from the anterior cerebral artery to share in supplying the right MCA territory [Figure 1b and d]. The aneurysm measured 6mm in size and was directed laterally. A right frontotemporal craniotomy was performed on the day of admission. After the dissection of the carotid cistern, we traced the right ICA and observed that the dup-MCA arose from the proximal ICA with an aneurysm arising from the trunk of these vessels [Figure 1c]. The dup-MCA projected toward the Sylvian fissure. With further retraction of the frontal lobe, aplasia of the main MCA was confirmed. The neck of the aneurysm was dissected and clipped successfully [Figure 1d]. The postoperative course was uneventful with no spasms or cerebral infarction.

## DISCUSSION

Intracranial vascular anomalies involving the MCA are relatively rare. Teal *et al*.[6] established a distinction between two types of acc-MCA. The vessel arising from the distal ICA between the anterior choroidal artery and the terminal bifurcation of the ICA and feeding the vascular territory of the normal MCA is referred to as a dup-MCA. On the other hand, a vessel that originates between the A1 and proximal A2 segments of ACA, reaching the sylvian fissure and feeding the territory of the MCA, is defined as an acc-MCA.[6,9] The coexistence of a dup-MCA with acc-MCA is relatively uncommon, accounting for approximately 0.2–4% of all angiographical findings. The prevalence of acc-MCA has been reported as 0.3–2.7%, while that of dup-MCA is 0.7–2.9% in autopsy- or angiography-based investigations. In our patient, the dup-MCA supplied the cortical territory of the temporopolar and the anterior or middle temporal arteries. The acc-MCA supplied the cortical territory of the orbitofrontal or prefrontal arteries.

Uchino *et al*.[7] diagnosed a larger MCA as a main MCA and a smaller MCA as an anomalous MCA. The dup-MCA has been reported to be smaller than the main MCA.[4] However, the main MCA and the dup-MCA occasionally had the same diameter, or the main MCA was smaller than the dup-MCA.[2,6,9] The carotid bifurcation is the junction of the ICA, main MCA, and ACA. We confirmed the aplasia of the main MCA based on 3D-CTAs and the intraoperative findings. In the present case, the hypoplastic dup-MCA supplied the cortical region of the temporopolar artery, and the acc-MCA supplied the cortical region of the MCA artery as a collateral blood supply to spare the aplastic main MCA artery. Therefore, the present report is the first case in which an ICA aneurysm arose from the trunk of a hypoplastic dup-MCA associated with acc-MCA and aplasia of the main MCA.

The embryologic explanation for anomalies and variations of the MCA remains unclear. The MCA develops after the ACA, and the ACA is considered a continuation of the primitive ICA. Thus, the MCA can be regarded as a branch of the ACA.[1] Embryologically, the MCA can be recognized in a 7–12mm embryo as twigs from the ICA proximal to the ACA. By the 16–18mm stage, the MCA has become more prominent and supplies branches that spread over the cerebral hemisphere. Yamamoto *et al*.[10] suggested that while an acc-MCA is a true anomalous artery, dup-MCA is instead a variation in the branching of the ICA.

The association between the dup-MCA, or the acc-MCA, and cerebral aneurysms has been well documented.[3,8] However, it is not clear whether this association is a chance occurrence or they are related by an unknown mechanism. In practice, it is important to preoperatively recognize such vascular anomalies so that the best surgical procedures for a cerebral aneurysm can be selected, since the collateral blood supply in patients with cerebral ischemia is associated with either dup- or acc-MCA. With respect to the clinical importance of dup-MCA and acc-MCA, the acc-MCA has the potential to serve as a collateral blood supply to the MCA territory in cases of MCA occlusion. The dup-MCA may also play an important role in supplying collateral blood flow to the frontal lobe and basal ganglia through the perforating arteries. The acc-MCA can be a collateral to the anterior frontal lobe, but it cannot supply flow with sufficient power to the main MCA territory.[5] Similarly, the dup-MCA can be collateral to the anterior temporal lobe, but it does not seem to supply enough blood to the main MCA territory. Komiyama *et al*.[4] reported that the dup-MCA has perforating arteries which attach it to the anterior perforated substance. Therefore, it is generally accepted that special attention should be paid to avoid ischemic complications and to find out whether a temporal clip should be used to disrupt the blood flow and thus control the premature rupture.

## References

[1] Abbie AA (1934). The morphology of the fore-brain arteries, with especial reference to the evolution of the basal ganglia. J Anat.

[2] Gibo H, Carver CC, Rhoton AL, Lenkey C, Mitchell RJ (1981). Microsurgical anatomy of the middle cerebral artery. J Neurosurg.

[3] Han DH, Gwak HS, Chung CK (1994). Aneurysm at the origin of accessory middle cerebral artery associated with middle cerebral artery aplasia: Case report. Surg Neurol.

[4] Komiyama M, Nakajima H, Nishikawa M, Yasui T (1998). Middle cerebral artery variations: Duplicated and accessory arteries. AJNR Am J Neuroradiol.

[5] Komiyama M, Nishikawa M, Yasui T (1997). The role of the accessory middle cerebral artery as a collateral blood supply. AJNR Am J Neuroradiol.

[6] Teal JS, Rumbaigh CL, Bergeron RT, Segall HD (1973). Angiographic demonstration of fenestrations of the intradural intracranial arteries. Radiology.

[7] Uchino A, Kato A, Takase Y, Kudo S (2000). Middle cerebral artery variations detected by magnetic resonance angiography. Eur Radiol.

[8] Uchino M, Kitajima S, Sakata Y, Honda M, Shibata I (2004). Ruptured aneurysm at a duplicated middle cerebral artery with accessory middle cerebral artery. Acta Neurochir (Wien).

[9] Umansky F, Dujovny M, Ausman JI, Diaz FG, Mirchandani HG (1988). Anomalies and variations of the middle cerebral artery: A microanatomical study. Neurosurgery.

[10] Yamamoto H, Marubayashi T, Soejima T, Matsuoka S, Matsukado Y, Ushio Y (1992). Accessory middle cerebral artery and duplication of middle cerebral artery - terminology, incidence, vascular etiology, and developmental significance. Neurol Med Chir (Tokyo).

